# 687. Zinplava Expert Retrospective Observations (ZERO) Study Findings on *Clostridioides difficile* Recurrence

**DOI:** 10.1093/ofid/ofad500.749

**Published:** 2023-11-27

**Authors:** Micah Jacobs, Sharon Morris

**Affiliations:** Romano, Pontzer and Assoc, Pittsburgh, Pennsylvania; Romano, Pontzer and Assoc., Pittsburgh, Pennsylvania

## Abstract

**Background:**

Describe real world experience in the use of bezlotoxumab (BZT) for the prevention of recurrent *Clostridioides difficile*(CDI) infection with a 1 year follow up following administration. The original BZT studies showed approximately 16% rate of recurrence at 12 weeks with other baseline and outcome data as noted in Figure 1.

**Figure:**
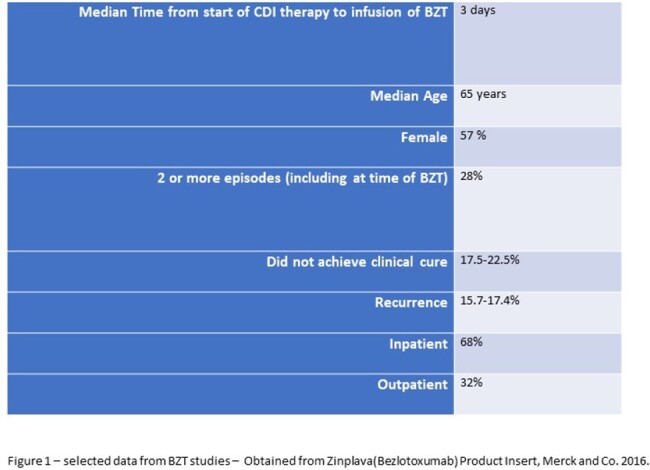

**Methods:**

Retrospective study of patients administered BZT in a community based private practice infusion suite from May 2017 to October 2020. A total of 50 consecutive patients were included with background demographics, relevant medical history and CDI history obtained through chart review. In addition to the chart review, patients were contacted at least 1 year after BZT administration to see if there were any recurrences and if other treatments were required. Primary objective was to show the rate of CDI recurrence in the 1 year after BZT administration. All BZT was administered in an outpatient setting in patients who were responding to initial Standard of care (SOC) therapy.

**Results:**

50 patients were included with infusion dates from May 2017 to October 2020. Baseline patient characteristics (Figure 2) include median Age of 69 and 74% female. CDI history (Figure 3) includes an average of 2.06 episodes including the current episode at time of time treatment and median of 19.5 days to BZT administration from last positive episode (positive test or starting a new treatment course for presumed positive). SOC treatments over the course of patient’s episodes are listed in Figure 3, most patients received more than agent.

In the 1 year following BZT only 4 patients had recurrence (Figure 4) with 2 going on fecal microbiota transplant. There were 3 deaths reported, all at least 3 months after BZT and none had CDI recurrence.

**Figure:**
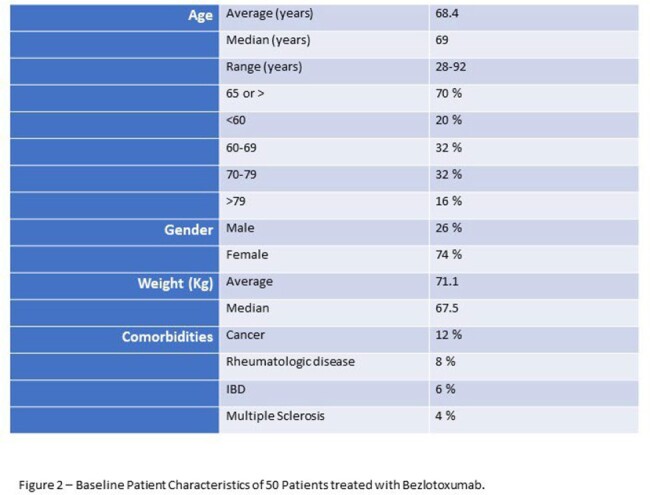

**Figure:**
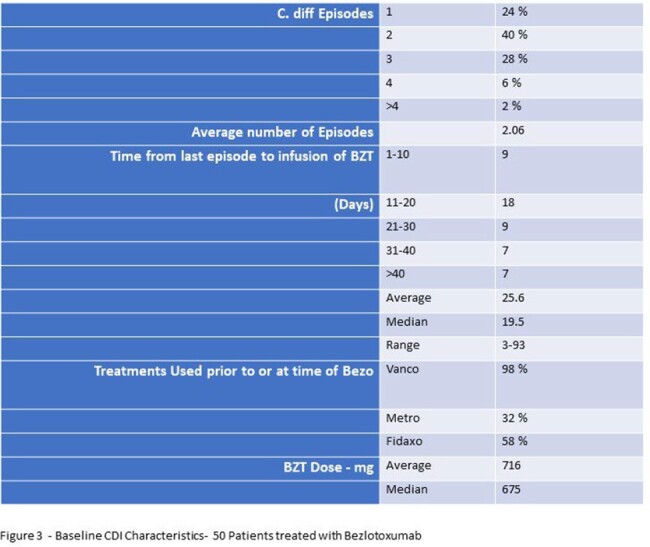

**Figure:**
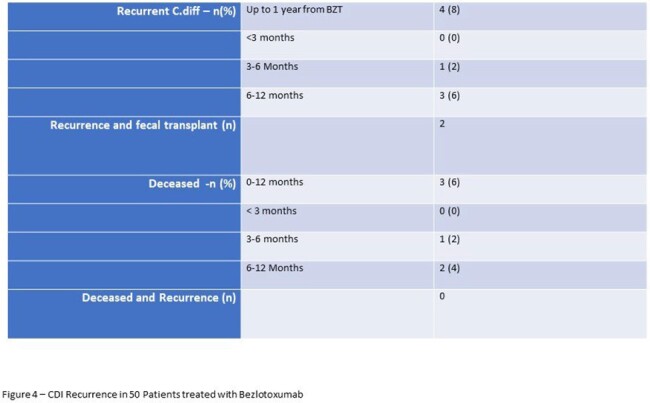

**Conclusion:**

In our real-world use, BZT administered in an outpatient setting to patients who responded to SOC treatment resulted in only 8% recurrence after 1 year compared to 16% after 12 weeks in the trial. This supports the existing guidelines on the use of BZT in patients over 65 and with recurrent CDI to prevent further recurrence. Our data also supports administration later in the course of therapy when patients have achieved initial response.

**Disclosures:**

**MICAH JACOBS, Micah Jacobs, MD, FIDSA, FACP**, Abbvie: Honoraria|Cumberland Pharmaceuticals: Honoraria|Merck: Grant/Research Support|Spero Pharmaceuticals: Advisor/Consultant

